# A Computational Model for Pain Processing in the Dorsal Horn Following Axonal Damage to Receptor Fibers

**DOI:** 10.3390/brainsci11040505

**Published:** 2021-04-16

**Authors:** Jennifer Crodelle, Pedro D. Maia

**Affiliations:** 1 Department of Mathematics, Middlebury College, Middlebury, VT 05753, USA; 2 Department of Mathematics, University of Texas at Arlington, Arlington, TX 76019, USA; pedro.maia@uta.edu

**Keywords:** pain processing, dorsal horn, neuronal dynamics, computational model, axonal damage, painful neuropathies

## Abstract

Computational modeling of the neural activity in the human spinal cord may help elucidate the underlying mechanisms involved in the complex processing of painful stimuli. In this study, we use a biologically-plausible model of the dorsal horn circuitry as a platform to simulate pain processing under healthy and pathological conditions. Specifically, we distort signals in the receptor fibers akin to what is observed in axonal damage and monitor the corresponding changes in five quantitative markers associated with the pain response. Axonal damage may lead to spike-train delays, evoked potentials, an increase in the refractoriness of the system, and intermittent blockage of spikes. We demonstrate how such effects applied to mechanoreceptor and nociceptor fibers in the pain processing circuit can give rise to dramatically distinct responses at the network/population level. The computational modeling of damaged neuronal assemblies may help unravel the myriad of responses observed in painful neuropathies and improve diagnostics and treatment protocols.

## 1. Introduction

Pain is the most common patient complaint in medical consultations and an experience that we will all have at some point during our life [1]. Our pain-processing mechanism emerges from a complex interplay of cognitive and affective processes with neurochemical and neuroanatomic systems [2]. The dorsal horn (DH) is an area of the spinal cord that plays a central role in processing nociceptive, or painful, signals with the midbrain and cortex providing top-down modulation to that circuitry [3]. As a consequence, this region is a common target for analgesic action and is thought to undergo changes that contribute to the exaggerated pain felt after nerve injury and inflammation [4]. For all its significance, the heterogeneity of various neuronal components of the dorsal horn circuitry continues to challenge our understanding of how we process painful sensory information.

Over the years, computational models of the neural activity associated with the DH gave rise to key insights regarding nociception-processing [5,6,7,8]. Most models posit that nociceptive activity is inhibited by Aβ-fibers unless the activity in the C-fibers (painful stimuli) outweighs it, thus activating the peripheral nerves and create the experience of pain. While this gate control theory greatly simplifies the physiological mechanisms of pain processing [9,10,11], it does provide a reasonable starting point for most computational and modeling efforts. In this study, we use the model introduced by Crodelle et al. [12,13] as a platform to numerically simulate the population activity of projection, inhibitory, and excitatory neurons in the DH under healthy and pathological conditions.

The dynamics of injured neuronal assemblies is a topic of broad and current interest in computational neurology, with axonal damage and their distortions to the neural activity being a hallmark feature of traumatic brain injuries and degeneration [14,15,16,17]. While there are multiple mechanisms associated with axonal damage (such as focal axonal swellings or demyelination), they invariably alter the usual transmission of spike trains along neuronal fibers. Maia et al. [14] assembled a list of phenomenological input/output rules describing commonly-observed forms of spike train distortions, which led to several studies simulating the addition of an injured-neuron population to functional networks [18,19,20,21,22,23].

Having a computational model capable of simulating a broad repertoire of outcomes from injured neurons in the DH may provide new insight into abnormal pain processing, a symptom present in fibromyalgia, peripheral neuropathic pain, and other pain-related disorders [24,25]. In what follows, we model the effects of axonal damage [14] to mechanoreceptor and nociceptor fibers in the pain processing circuit [12,13] and illustrate how different types of axonal dysfunction give rise to distinct responses at the network/population level.

## 2. Materials and Methods

### 2.1. Overview of Spinal Cord Model for Pain Processing

**Figure 1:**
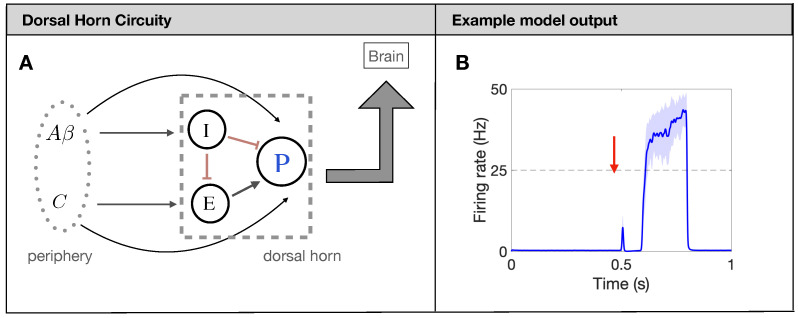
Firing-rate neural network model for pain processing. (**A**): Schematic of the spinal-cord circuitry. Projection neurons [*P* in (**A**)] receive input from excitatory (*E*) and inhibitory (*I*) interneurons, as well as directly from two types of afferent fibers: Aβ fibers and *C* fibers. (**B**): Typical output response (firing rate of *P* neurons in (**A**)) of a healthy network to a brief painful stimuli at 0.5 s.

In what follows, we utilize the firing-rate model for the processing of painful stimuli in the human spinal cord developed in [13]. The model is based on the widely-used gate-control theory of pain [9]. Shortly, projection neurons (*P* in Figure 1A) receive input from excitatory (*E*) and inhibitory (*I*) interneurons, as well as directly from two types of afferent fibers: Aβ fibers and *C* fibers. While there are many different types of afferent fibers carrying sensory information about touch, pressure, itch, burning, and pain, in this model, we focus on the nociceptive pain processing circuit and refer to all fibers carrying mechanical touch information as Aβ fibers and those carrying slow pain as *C* fibers, as has been done previously [5,13]. Each population (*P*, *E*, and *I*) responds to a weighted input of firing rates from input (presynaptic) populations as determined by individual nonlinear response curves in the formalism of [26,27]. The model also includes N-methyl-D-aspartate (NMDA) type synapses from the *C* fibers to the *P* neurons modeled as a modulation of the synaptic weight as a function of *P*-neuron firing rate. All model details can be found in [13] and Appendix A.

The underlying mechanism for the model to produce a prototypical pain response, see Figure 1B, is the timing of the input from the Aβ and *C* fibers. Due to factors like axon myelination and diameter, the afferent fibers have significantly different conduction speeds leading to a distribution of arrival times in the DH of about 0–20 ms for Aβ and 90–300 ms for *C* [28]. To capture this characteristic, we introduce the delay and distribution on the Poisson spike trains for each fiber so that the DH output matches the experiments (again, see Figure 1B). Throughout the simulation, each fiber also has a spontaneous background firing rate of 1 Hz [29]. Finally, we average the Poisson spikes generated on each fiber (380 Aβ fibers and 820 *C* fibers for a total of about 1200 as observed in one nerve bundle of rat [30,31]) over all fibers to yield an average firing rate for Aβ fibers and *C* fibers. This average firing rate for the fibers then serves as a weighted input to the nonlinear response functions of the DH populations. We measure the painful output of the model by quantifying characteristics of the response of the *P* neurons during the time at which the *C* fibers reach the DH (about 700 ms–750 ms), which will be further explained in Section 2.4.

### 2.2. Modeling Effects of Neuronal Injury to Spike-Train Activity

**Figure 2:**
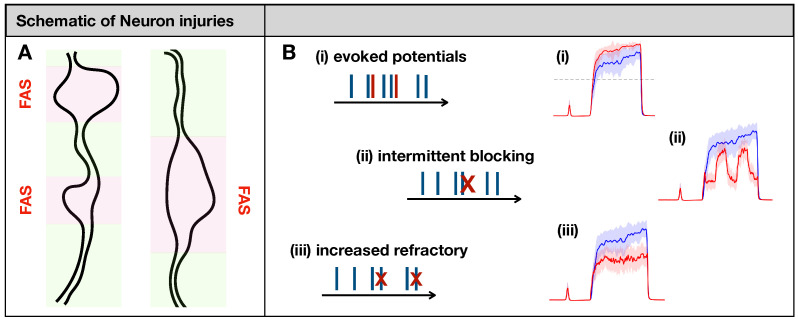
(**A**): Focal Axonal Swellings (FAS)—also called varicosities or beadings—are enlarged, heterogeneous structures along axonal shafts that may dramatically distort conduction and synaptic transmission (see [16,33,34] and references therein). In our schematics, normal axonal segments are indicated in green, while the FAS portions are highlighted in pink/red. FAS are the most common forms of neuronal injury that may follow mechanical trauma or neurodegenerative disorders. (**B**): Previous computational modeling work characterized different ways in which FAS may affect spike train propagation [14,15,16,17]. In this work, we will simulate axonal injury by incorporating FAS effects that lead to (i) creation and (ii–iii) deletion of spikes. At the population level, neuronal injuries may lead to dramatically different pain-processing responses (in red) compared to the healthy system (in blue).

Maia et al. [14] posited eight types of phenomenological input/output rules describing spike train distortions caused by the major forms of neuronal injury, including axonal swellings [15,16,17] and demyelination [32]. In their formulation, a spike train $\left\{x_{n}\right\}$ is transformed into another spike train $\left\{y_{n}\right\}$ according to some rule. If faithful conduction occurs, the input/output spike trains will match ($y_{n}=1\cdot x_{n})$. In the case of severe injury, the axonal impairment will delete all spikes in the train ($y_{n}=0\cdot x_{n}$). While these two cases would lead to trivial outcomes at the population level, some other types of distortion would not (see Figure 2B):

(i) Evoking potentials: In this rule, a single input spike triggers the formation of *k* additional spikes.
(ii) Intermittent blocking: In this rule, the spike train switches between (total) blocking and normal conduction periodically (with period f=2π/ω).
(iii) Increasing refractoriness: In this rule, consecutive spikes may be deleted if the inter-spike interval between them is below τ. This effectively increases the refractory period of the spike train.

We note that the latter impairment is strongly frequency-dependent; spike trains with higher firing rates are more strongly affected than spike trains with lower firing rates. This will ultimately lead to confusions of higher firing rates by lower ones. For more details regarding the mathematical formulation and numerical implementation of these rules, see [14].

### 2.3. Injury Protocols for Receptor Fibers

In this study, we opted to damage the receptor fibers (*C* and Aβ fibers) with the injured rules listed above. The goal of our injury protocols is twofold: (i) to illustrate a large variety of potential outcomes at the collective level, and (ii) to study the effects of each parameter of the system. For the *C*-fibers, we introduce injured neurons to its population that distorts spike trains by evoking potentials, blocking spikes intermittently, and by increasing their refractoriness. For the Aβ fibers we introduce injured neurons that delay their spike trains by $d^{⋆}$ ms. In our first simulations, we target the fiber populations separately, but in the last one, we consider a “mixed-effect” protocol where damaged *C*-fibers evoke potentials (that increases pain) concomitantly with spike train delays in the Aβ fibers (that reduces pain). This creates a “tug-of-war” between the injuries, and a state in which minor fluctuations could lead to significant oscillations in pain perception.

### 2.4. Quantitative Markers for Pain Response

**Figure 3:**
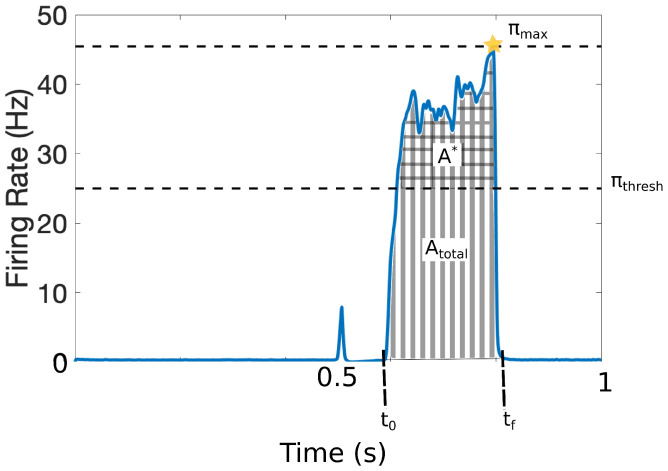
Quantitative markers for pain response: $\pi_{\mathrm{thresh}}$ = 25 Hz represents the firing-rate threshold for painful responses; $A^{*}$ (checked area) is the total area above that threshold, $A_{Total}$ (striped area) denotes the total area of the pain response (including above $\pi_{\mathrm{thresh}}$); $t_{0}$ defines the initial time of pain response and $t_{f}$ the final time of the pain response; and $\pi_{max}$ denotes the maximum achieved firing rate of the pain response. We also define the average firing-rate response, $\pi^{*}=A^{*}/\left|t_{f}-t_{0}\right|$ and the number of times $N_{C}$ that the signal crosses the pain threshold ($N_{C}=2$, not depicted).

It is challenging to quantify pain in a clinical setting since it involves cognitive and affective processes along with the patients’ subjective assessments. To avoid ambiguities in this work, we characterize the pain response with the set of quantitative markers illustrated in Figure 3. The output of the model is the response that the projection neurons integrate from the DH and transmit to the cortex within the time interval $\left[t_{0},t_{f}\right]$. The model was calibrated such that $\pi_{\mathrm{thresh}}$ = 25 Hz represents the firing-rate threshold for painful responses [12,13]. Figure 3 shows a typical painful response and depicts the following quantitative markers:

$$\begin{array}{ccc} A_{Total} & = & \mathrm{Total}\;\mathrm{area}\;\mathrm{under}\;\mathrm{the}\;\mathrm{curve}\\ A^{*} & = & \mathrm{Area}\;\mathrm{above}\;\;\pi_{\mathrm{thresh}}\\ \pi^{*} & = & \mathrm{Average}\;\mathrm{firing}\;\mathrm{rate}\;\mathrm{response},\;\;\pi^{*}=A^{*}/\left|t_{f}-t_{0}\right|\\ \pi_{max} & = & \mathrm{Maximum}\;\mathrm{achieved}\;\mathrm{firing}\;\mathrm{rate}\\ N_{C} & = & \;\mathrm{number}\;\mathrm{of}\;\mathrm{times}\;\mathrm{the}\;\mathrm{painful}\;\mathrm{response}\;\mathrm{crosses}\;\pi_{\mathrm{thresh}} \end{array}$$

The addition of injured fibers to the neuronal population may significantly alter the overall pain response and consequently, these four quantitative pain markers.

## 3. Results

### 3.1. Effects of Different Injuries on C Fibers

**Figure 4:**
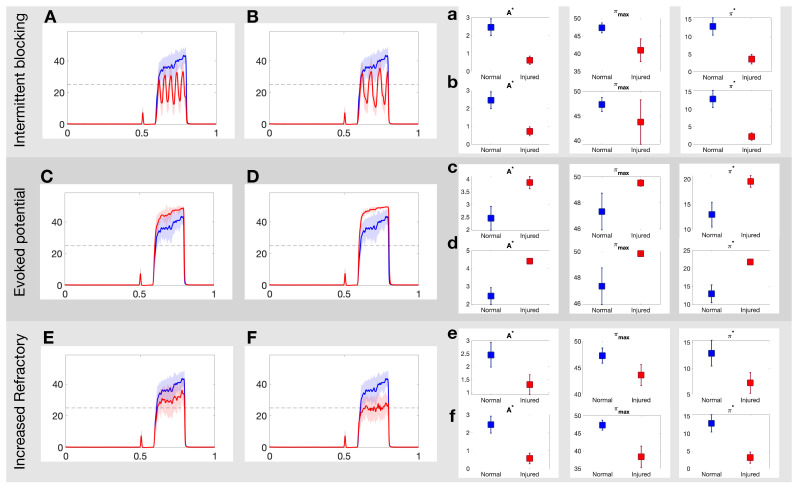
Example realizations of the DH projection neuron output to each of the three injuries on the C-fibers. (**A**,**B**): Projection neuron firing rate in response to intermittent blocking injury with frequency of π/20 and π/40, respectively; (**C**,**D**): evoked potential injury for an evoked probability of 20% and 40%, respectively; and (**E**,**F**): increased refractory injury with a period of 15 ms and 25 ms, respectively. See Section 2.2 for more details about each injury. (**a**–**f**): Three quantitative measures area above threshold, $A^{*}$; average firing rate response, $\pi^{*}$; number of threshold crossings of firing rate response, $N_{C}$, for each injury for normal (blue) and injured (red) responses in the projection neurons. See Section 2.4 for more details on each quantitative pain marker.

Figure 4 illustrates how the painful response changes when populations of injured C fibers are introduced into the system. In these simulations, the injured neurons are all of the same type, i.e., we consider only one input/output rule to govern spike train distortion at a single time. The top panels in Figure 4 show the effect of the intermittent blocking injury with frequency of π/20 (A) and π/40 (B), respectively. In both cases, the switches between (total) blocking and normal conduction within the spike trains lead to oscillations in the overall response. The maximum achieved firing rate ($\pi_{\max}$), the area of the curve above 25 Hz ($A^{*}$) and the average firing rate response ($\pi^{*}$) all decay after injury. The injured responses also appear to be more irregular than the stereotypical response. The mid panels in Figure 4 show a contrasting case, where the pain response increases due to the evoked potentials (which may occur with a probability of 20% and 40% in Panels C and D, respectively). In this case, $\pi_{\max},A^{*}$ and $\pi^{*}$ increase proportionally to the evoked probability. Finally, the bottom panels in Figure 4 show the effects of the increased refractoriness injury type. In this rule, a consecutive spike is deleted if it’s within 15 ms (Panel E) or 25 ms (Panel F) from the preceding spike, and as a consequence, all quantitative pain markers also decay.

**Figure 5:**
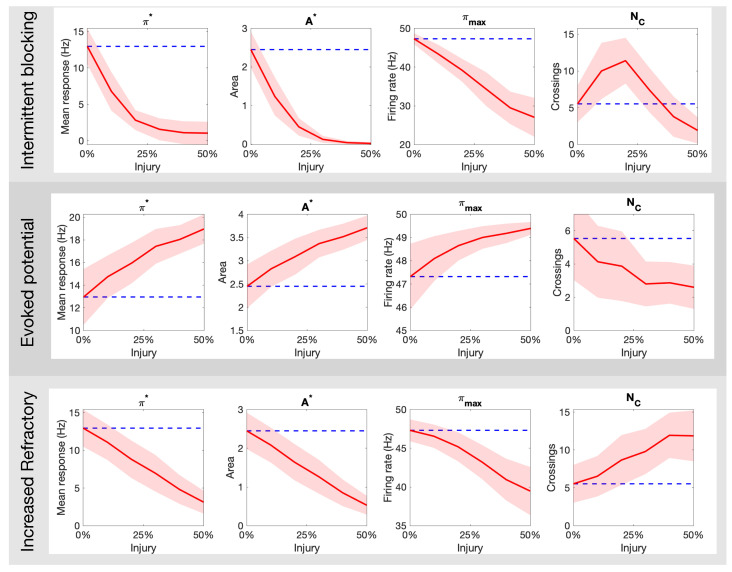
How the different pain quantitative markers ($\pi^{*},A^{*},A^{0},\pi_{\max}$ and $N_{C}$) change as a function of the type (intermittent blocking, evoked potentials, and increased refractory) and amount (% of *C* fibers damaged) of neuronal damage in the C-fibers. The blue dashed line indicates the mean response for a healthy population.

Figure 5 expands on the qualitative results above by quantifying the markers for pain response as a function of the percentage of injured neurons in the targeted fiber population. The top panel of Figure 5 shows the result for intermittent blockage (with period f=2π/10); we observe a monotonic decay in the parameters $\left[\pi^{*},A^{*},A^{0},\pi_{\max}\right]$, but a maximum $N_{C}$ value is achieved near 25% injury level. The middle panel of Figure 5 shows analogous plots for the increased refractoriness injury (with τ=10 ms). The markers follow a similar trend with the exception of $N_{C}$ that seems to exhibit a sigmoid-like shape. These two forms of injury contrast dramatically to the evoked potential injury shown in the bottom panel of Figure 5. There, the parameters $\left[\pi^{*},A^{*},A^{0},\pi_{\max}\right]$ increase monotonically while $N_{C}$ decays. Overall, these results show that different types of axonal injuries in the *C*-fibers can lead to dramatic differences in all quantitative markers of pain.

### 3.2. Optimal Spike Delays Parameters for Aβ Fibers

In this section, we target the second type of afferent fiber with our injury protocols. Due to myelination and larger diameter size, the conduction speed in the Aβ fibers is much faster than in the *C* fibers, and any stimulation of Aβ fibers will reach the dorsal horn about 100 ms before the response from stimulation of the C fibers. Since we measure pain with respect to the activity of the projection neurons during the C response (recall Figure 3), the most likely way for injured activity on the Aβ fibers to affect the C response is to slow down the conduction speed or delay the spikes.

**Figure 6:**
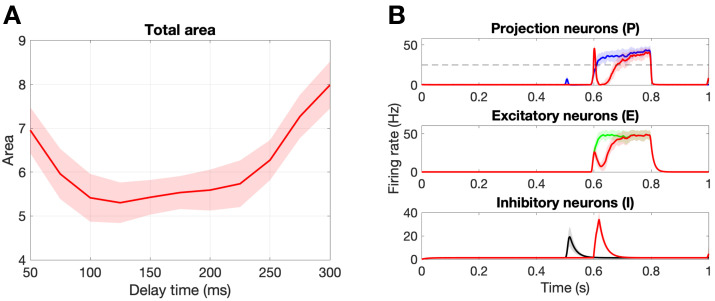
Damaging the Aβ fibers. (**A**): Damaging 50% of the Aβ fibers with different delay values to find the optimal delay time, $d^{⋆}$ = 125 ms, that yields the minimal total area. Note that no C fibers were damaged and we are calculating the total area $A_{\mathrm{total}}$. (**B**): Population responses of the projection, excitatory, and inhibitory neurons. The colored curves represent a healthy response, while the red curves represent the damaged response for the optimal delay time $d^{⋆}=125$ ms. Note the shaded region is the standard deviation over 30 realizations.

Figure 6A shows the change in the total area marker, A${}_{\mathrm{total}}$, for delay times from 50 ms to 300 ms. First, we point out that there exists an optimal delay time of $t^{⋆}=125$ ms that minimizes the total area, $A_{\mathrm{total}}$. Figure 6B shows the population responses for this optimal delay parameter as compared to a healthy response. Notice that stimulation on the Aβ fibers during the C response (∼600–800 ms) leads to an inhibition of the firing rate of the projection neurons due to the circuitry suggested by gate control theory. Namely, that Aβ fibers synapse onto the inhibitory interneurons, which in turn synapse onto the projection neurons. In the original paper by Crodelle et al. [13], this is the mechanism by which the phenomenon known as pain inhibition works. The amount of pain inhibition depends on the timing of the Aβ stimulation, with optimal pain relief found for time delays that allow the Aβ pulse to occur near the beginning of the C response, as is the case for $t^{⋆}=125$ ms.

Now that we established how pain might be affected by damaging the Aβ fibers, we go on to investigate possible interactions between damage on the Aβ fibers and on the C fibers.

### 3.3. Damaging Both Aβ and C Fibers

From Section 3.1, we saw that intermittent blocking and increased refractory in C-fibers both led to decreases in the response of the projection neurons, while evoked potentials led to an increased response. Since damaging the Aβ fibers (at particular delay times) also led to a decrease in the projection neuron response, it is redundant to consider the influence of two mechanisms to decrease pain. Instead, we consider evoked potentials on the C fibers so as to study the interplay between an increased response due to the C fibers and the pain inhibition evoked on the Aβ fibers due to delayed spike damage.

**Figure 7:**
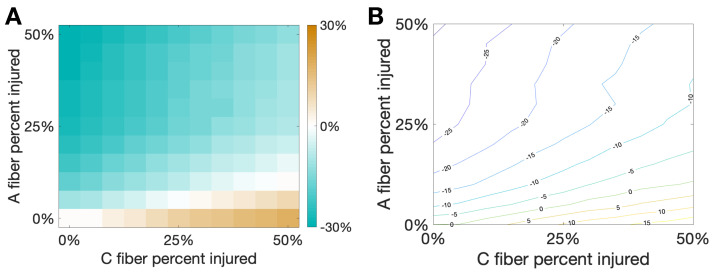
Damaging both Aβ and *C* fibers. In this simulation we consider a hybrid injury protocol in which signals from Aβ fibers are delayed (with delay $d^{⋆}=125$ ms) while evoked potentials are added to signals from *C* fibers (with evoked probability of 10%). This creates a “tug-of-war” between the two injury types that would respectively decrease/increase the pain marker $A_{\mathrm{total}}$. The horizontal/vertical axes correspond to the percentage of damaged fibers from each type. (**A**): Heat map and (**B**): Contour curves are shown, with color indicating the fraction percent change of $A_{\mathrm{total}}$ from the baseline pain response. Note that white regions in (**A**) and the contour curve associated to zero in (**B**) indicate injury combinations that would lead to the same $A_{\mathrm{total}}$ observed in baseline, with one type of injury effectively cancelling out the other.

Figure 7 shows the percent change of total area from the healthy case to the injured case for varying percentages of damaged C and Aβ fibers. We note that there is a sort of “tug-of-war” between the increased activity due to the evoked potentials on the C fibers and the pain inhibition elicited by the delayed spikes on the Aβ fibers. Interestingly, at the boundary of these two behaviors, illustrated by the white region in the left plot and the green curve in the contour plot, there is a balance between elicited spikes and spike inhibition such that there is no change in total area due to the different injuries on the two different fibers. This seems to suggest that small oscillations in the percentage of fibers injured near the boundary could potentially lead to oscillations in the observed pain response between increased pain and pain relief.

## 4. Discussion and Conclusions

### 4.1. Overview of Results

In this study, we use the computational model introduced by Crodelle et al. [12,13] to simulate pain processing in the dorsal horn under healthy and pathological conditions. Figure 1A shows the underlying circuitry in which C fibers transmit pain signals from the peripheral to the spinal cord while Aβ fibers transmit touch stimuli. In normal conditions, projection neurons would integrate the input signals on the dorsal horn and transmit a stereotypical pain response to the cortex (Figure 1B). This outcome can change dramatically, however, if spike-train distortions associated with axonal damage [14,15,16,17] are introduced in the system (see Figure 2). In Figure 3, we define five quantitative pain-markers $\left[A_{\mathrm{total}},A^{*},\pi^{*},\pi_{\max},N_{C}\right]$ to characterize the observed changes in pain response caused by different injury protocols. Figure 4 and Figure 5 show our results for when C-fibers are targeted with three different types of axonal injury (intermittent blocking, evoked potentials, and increased refractoriness). Figure 6 demonstrates another path for pain desensitization, where Aβ fibers are targeted and have their spike-trains delayed. Finally, in Figure 7 we consider a “tug-of-war” scenario where damaged C fibers increase the painful signal (by evoked potentials) while damaged Aβ fibers contribute in the opposite direction (by spike-train delays).

Our main goal throughout this work was to illustrate how a variety of non-trivial outcomes may emerge at the collective level from different types of injury protocols. Axonal damage in the C-fibers leading to evoked potentials/increased refractoriness translated to monotonic increase/decrease of all quantitative pain markers (Figure 4 and Figure 5). However, the $N_{C}$ marker (that counts the number of times the pain signal crosses the typical $\pi_{\mathrm{thresh}}\;=\;25$ Hz threshold) behaves very differently for the intermittent blocking injury (see top-right plot in Figure 5). In this kind of injury, the spike trains are blocked intermittently, which leads to an oscillatory-kind of collective response (see Figure 4B). If the percentage of injured neurons is small(large), the oscillations remain above(below) the π=25 Hz pain threshold, which in both cases lead to small $N_{C}$ counts. For an intermediate percentage, however, $N_{C}$ will achieve its maximum value. We posit that intermittent blocking and multiple crossings of pain threshold might be linked with throbbing/pulsating types of pain. Moreover, this feature does not require large percentages of injured neurons to be significant, which might explain its prevalence in clinical settings.

Pain desensitization can also be achieved, in a more subtle way, by targeting Aβ fibers. Specifically, we demonstrated that the occurrence of spike-train delays in Aβ fibers will derange the expected response of the inhibitory neurons, which in turn, will lead to an overall weaker pain signal. Figure 6 shows that a delay of $d^{⋆}=125$ ms leads to a minimal area under the curve ($A_{\mathrm{total}}$) in the pain response. We conclude our exploration by creating a “tug-of-war” scenario in which the pain desensitization caused by these damaged A-fibers competes with the sensitization caused by damaged C-fibers (that evoke potentials). As shown in Figure 7, these effects can cancel each other out (in the white boundary-like region between regimes). We conjecture that this delicate balance between the regimes could be offset, for instance, by fluctuations in the patient’s circadian rhythm. In particular, healthy subjects typically experience the highest sensitivity to pain in the middle of the night and lowest in the afternoon, while patients suffering from neuropathy experience an approximately 12-h shift in their rhythmicity [12]. Altogether, improvements in the knowledge of the transmission of pain from the damaged nerve to the pain-processing center in the spinal cord may lead to better diagnostics and treatment protocols.

### 4.2. Connection to Neuropathic Pain

Our framework may provide insights into neuropathic pain, a form of pain-disorder that affects 7%–10% of the general population in which the underlying pathophysiology remains a contested topic [35]. Lesions on the peripheral fibers (Aβ, Aδ and *C* fibers) are linked to distortions of sensory signals into the spinal cord and the brain [35,36]. One piece of the puzzle unraveled by micro-neurography studies is the presence of ectopic activity in primary afferent fibers [37,38]. If an afferent fiber is disconnected from the periphery due to an injury or a lesion (for example, neuroma C fiber afferents), the remnants can generate ectopic activity [39] while the intact fibers may become hyperexcitable [40]. The enhanced excitability of spinal neurons produces may enable afferent fibers to activate second-order nociceptive neurons, generating the so-called central sensitization [41,42]. Thus, incorporating injured neuronal populations into the computational models of pain processing might shine new light on different types of stimulus-evoked pain (hyperalgesic vs. allodynic) or painful neuropathies associated with other diseases [43,44].

### 4.3. Limitations and Future Work

The modeling effort presented here represents a high-level overview of potential mechanisms that might underlie the observed effects of axonal injury on pain experience. The study has several limitations. First, the circuitry in the spinal cord is much more complicated than presented here (see [45] for a review) and is still being unraveled [4,46]. In addition, the gate control theory of pain is flawed and doesn’t account for many observed phenomena [10,11], although many models use it as a basis for spinal circuitry [5,6,8]. The population-level firing-rate model used in this work does not reflect the reality that DH neurons are heterogeneous and exhibit distinctive action potential shapes and sizes, as well as different ion distributions. However, as shown in [13], the model uses distinct response curves for each population motivated by experiments and includes N-Methyl-D-Aspartate (NMDA)-like synapses by modeling the weight to the projection neurons as a function of the projection neuron activity. As a result, this simplified spinal-cord model can replicate known experimental pain phenomena such as pain inhibition and wind up.

Finally, our injury protocols should be regarded as a proof-of-concept at this stage since a single type of axonal injury (per fiber population) is unrealistic; Maia et al. [21], for instance, reported different types of focal axonal swelling distributions (which included normal transmission, filtering, reflection, and total blockage regimes). Other neuronal subpopulations (besides C fibers and Aβ fibers) may also be dysfunctional, which could lead to more sophisticated mixed effects and tugs-of-war. In future work, we expect to gather more experimental evidence/parameters to calibrate the model more realistically for different pain disorders. All things considered, the computational modeling of damaged neuronal assemblies is a promising avenue that may help unravel the myriad of responses observed in painful neuropathies.

## Data Availability

All significant MATLAB codes are made available at https://github.com/jcrodelle/damagedFibers (accessed on 15 April 2021).

## Abbreviations

The following abbreviations are used in this manuscript:

DH	Dorsal Horn
FAS	Focal Axonal Swelling

## Appendix A. More Details on the Spinal-Cord Model

Here we present more details on the firing-rate model used in this work for pain processing in the spinal cord, a simplified version of the model developed in [13]. We assume that the average firing rate of the projection, inhibitory, and excitatory neuron populations, $f_{P}$, $f_{I}$, and $f_{E}$, respectively, follow the dynamic equations

(A1) $$\begin{array}{ccc} \frac{df_{P}}{dt} & = & \frac{P_{\infty }\left(g_{A\beta P}f_{A\beta }\left(t\right)+g_{A\delta P}f_{A\delta }\left(t\right)+\left(g_{\mathrm{CP}}+g_{\mathrm{NMDA}}\right)f_{C}\left(t\right)+g_{\mathrm{EP}}f_{E}-g_{\mathrm{IP}}f_{I}\right)-f_{P}}{\tau_{P}}\;,\\ \frac{df_{E}}{dt} & = & \frac{E_{\infty }\left(g_{\mathrm{CE}}f_{C}\left(t\right)-g_{\mathrm{IE}}f_{I}\right)-f_{E}}{\tau_{E}}\;,\\ \frac{df_{I}}{dt} & = & \frac{I_{\infty }\left[g_{A\beta I}f_{A\beta }\left(t\right)\right]-f_{I}}{\tau_{I}}\;, \end{array}$$

where *t* is time in seconds, and $\tau_{P}=0.001$ s, $\tau_{E}=0.01$ s, and $\tau_{I}=0.02$ s are the intrinsic time scales of each population. The populations communicate via average synapses that we refer to as weights $g_{ij}$ from presynaptic neuron populations *i* (i= Aβ, Aδ, C, P, E, I) to neuron population *j* (j= P, E, I).

We also include N-methyl-D-aspartate (NMDA) type synapses from the C-fibers to the P population through modification of the synaptic weight, $g_{\mathrm{NMDA}}$ by the average firing rate of the P population

(A2) $$\frac{dg_{\mathrm{NMDA}}}{dt}=\frac{M_{\infty }\left(f_{P}\right)-g_{\mathrm{NMDA}}}{\tau_{\mathrm{NMDA}}}\;,$$

where $\tau_{\mathrm{NMDA}}=1$ s.

The nonlinear response function for each population has the form

$$y_{\infty }\left(x\right)=\max\left(y\right)\frac{1}{2}\left[1+\tanh\left(\frac{1}{\alpha_{y}}\left(x-\beta_{y}\right)\right)\right],\;\;y=P,E,I,M,$$

mimicking the F-I curves found in [5]. Finally, for all pieces of the model, we use the same parameter values as in the original work [13].
